# Supplementary figures and images for: Interaction between Teneurin-2 and microtubules via EB proteins provides a platform for GABAA receptor exocytosis
Source: eLife. 2023 Jun 5;12:e83276. doi: 10.7554/eLife.83276 (PMC10284602; doi:10.7554/eLife.83276)

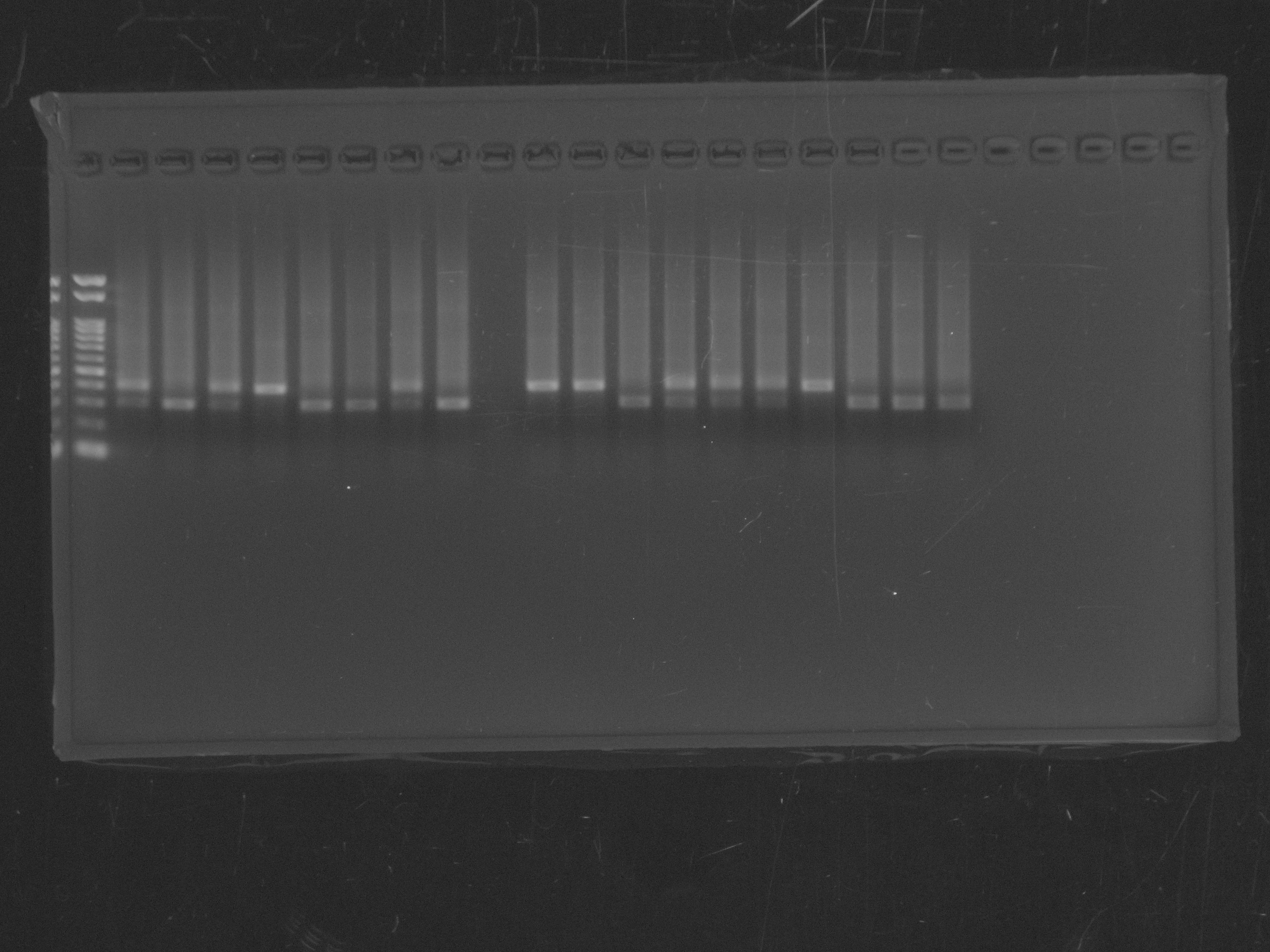

Supplement: Figure 3—figure supplement 1—source data 1. [file elife-83276-fig3-figsupp1-data1.zip › full_unedited_gels_1C.JPG]

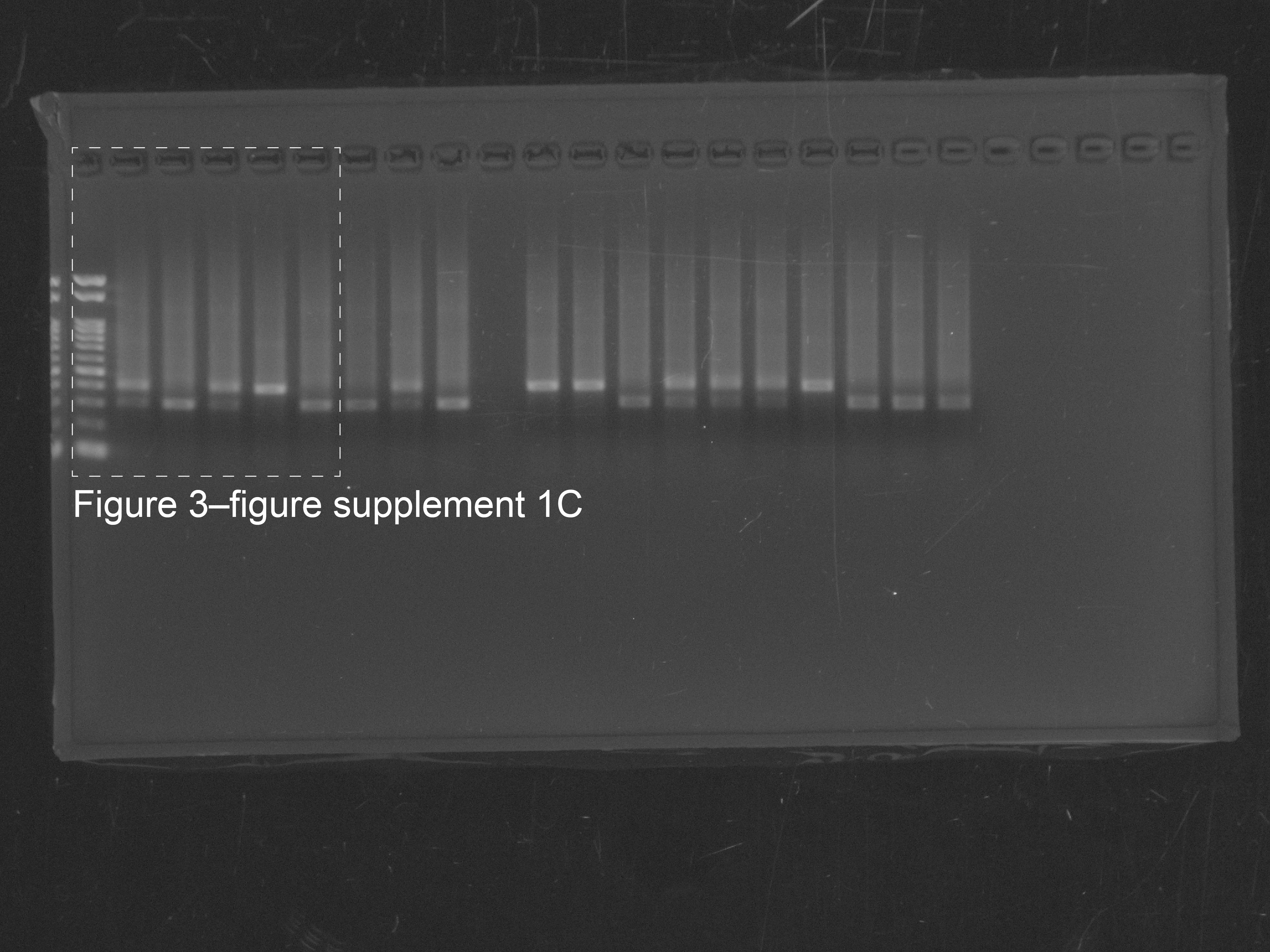

Supplement: Figure 3—figure supplement 1—source data 1. [file elife-83276-fig3-figsupp1-data1.zip › full_unedited_gels_with_label_1C.jpg]

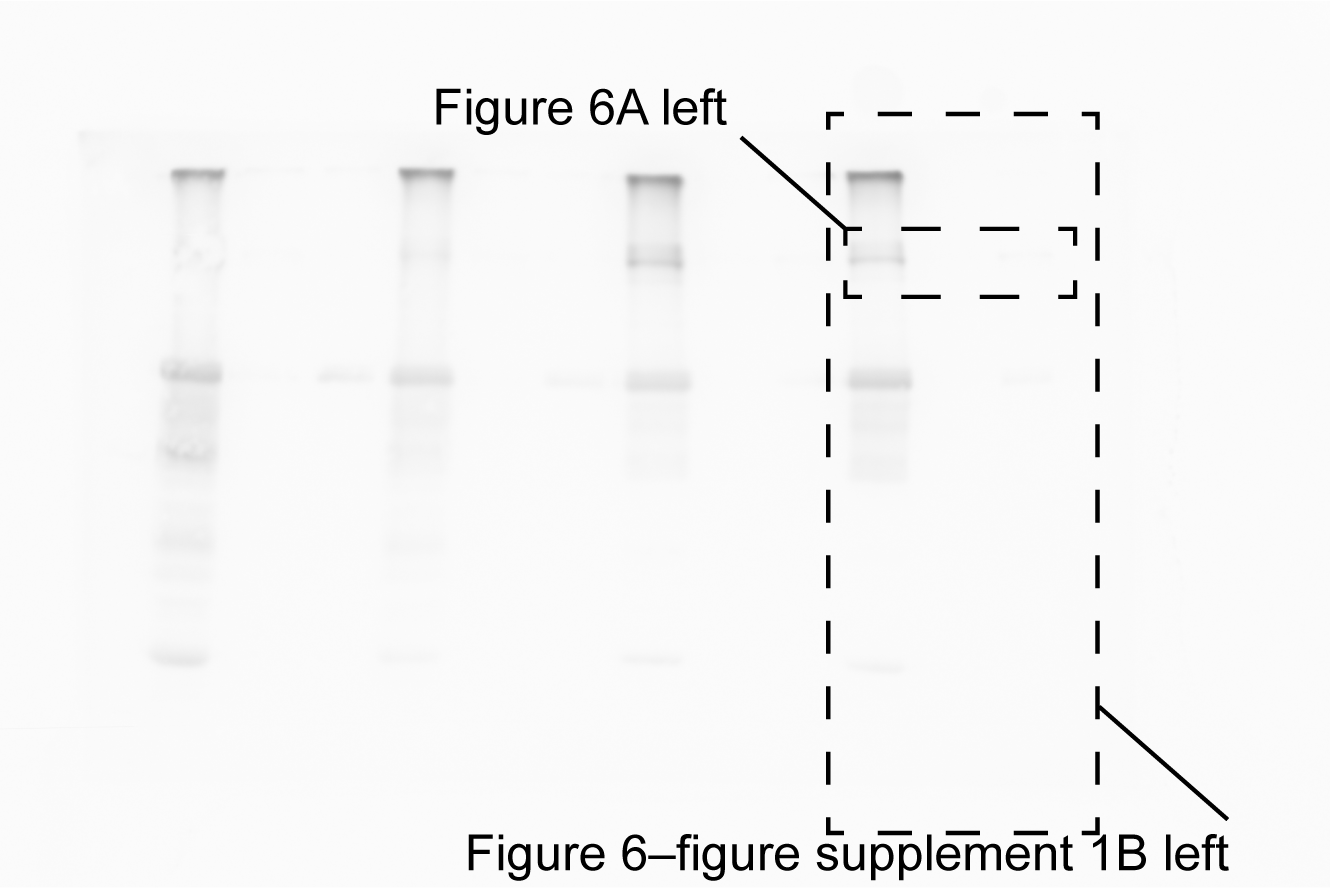

Supplement: Figure 6—source data 1. [file elife-83276-fig6-data1.zip › full_unedited_blots_with_labels_6A_left.tif]

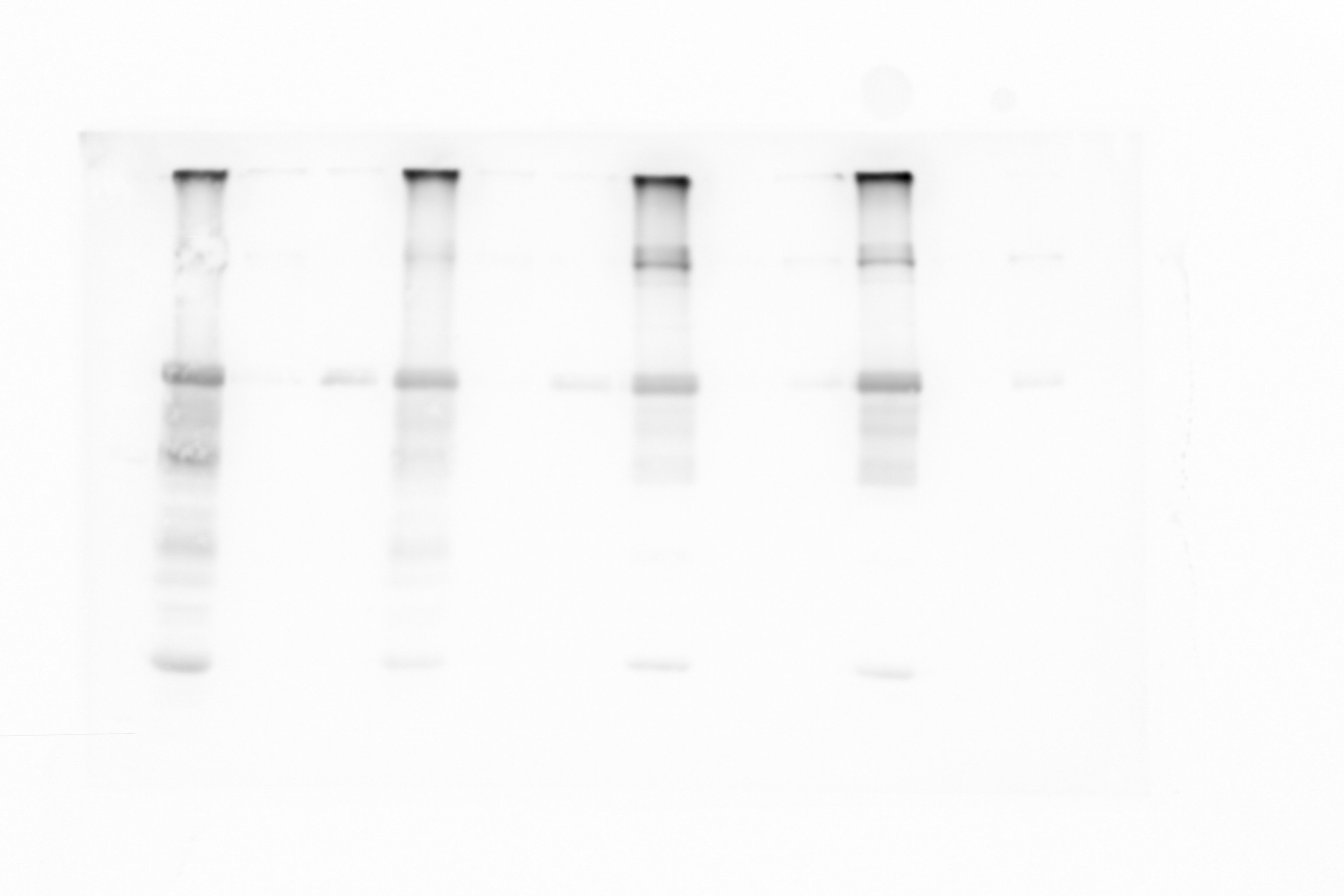

Supplement: Figure 6—source data 1. [file elife-83276-fig6-data1.zip › full_unedited_blots_6A_left.tif]

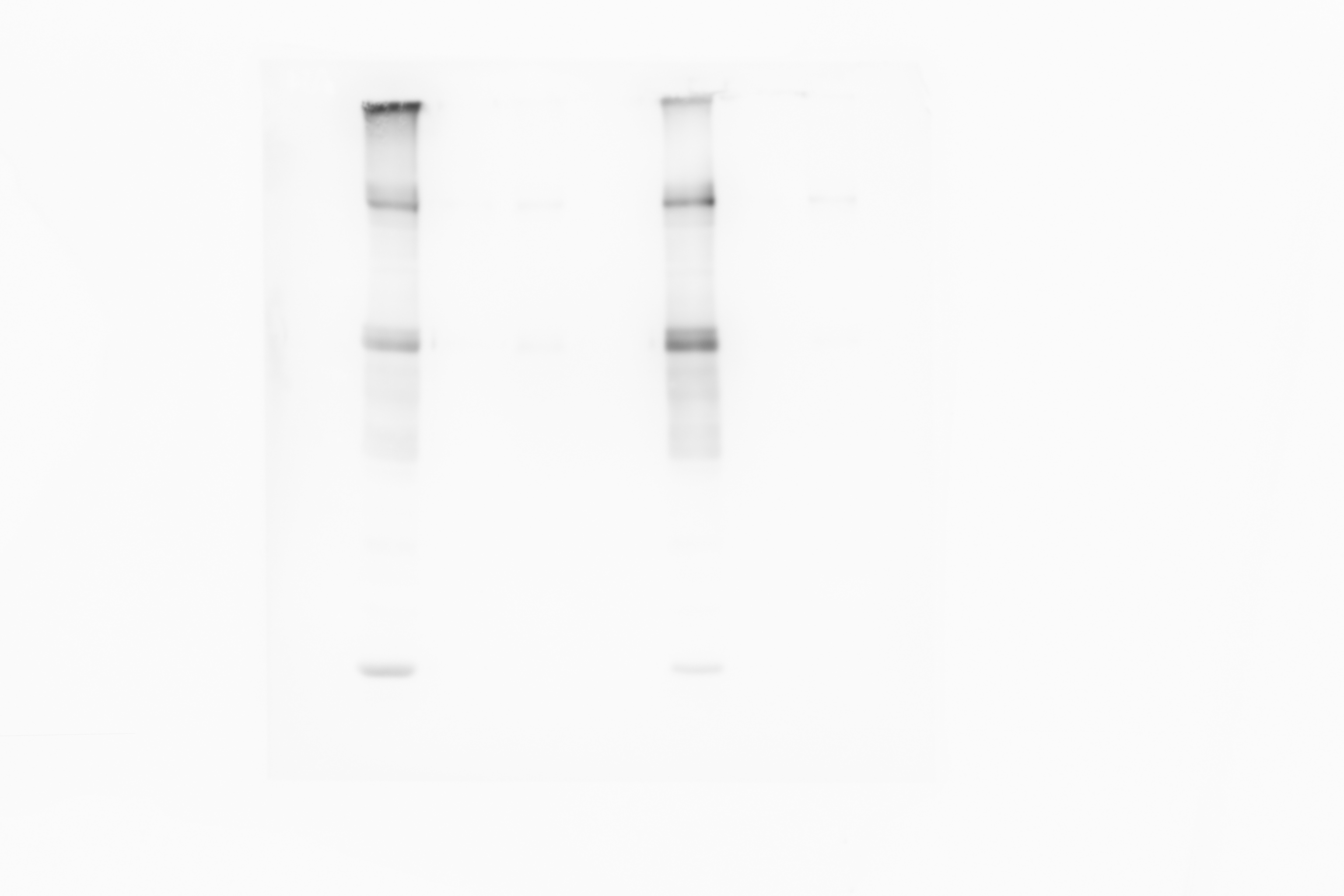

Supplement: Figure 6—source data 1. [file elife-83276-fig6-data1.zip › full_unedited_blots_6A_right.tif]

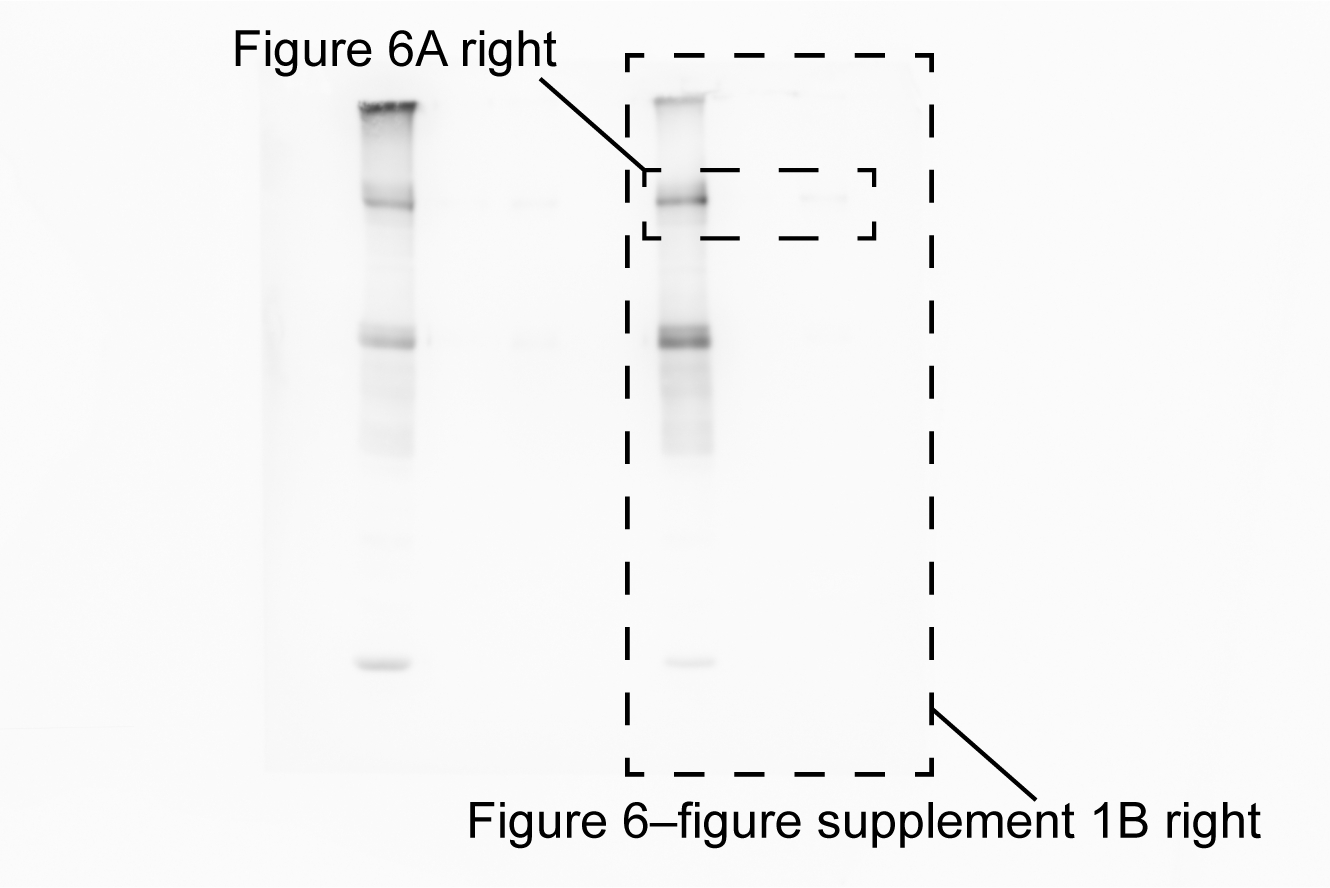

Supplement: Figure 6—source data 1. [file elife-83276-fig6-data1.zip › full_unedited_blots_with_labels_6A_right.tif]

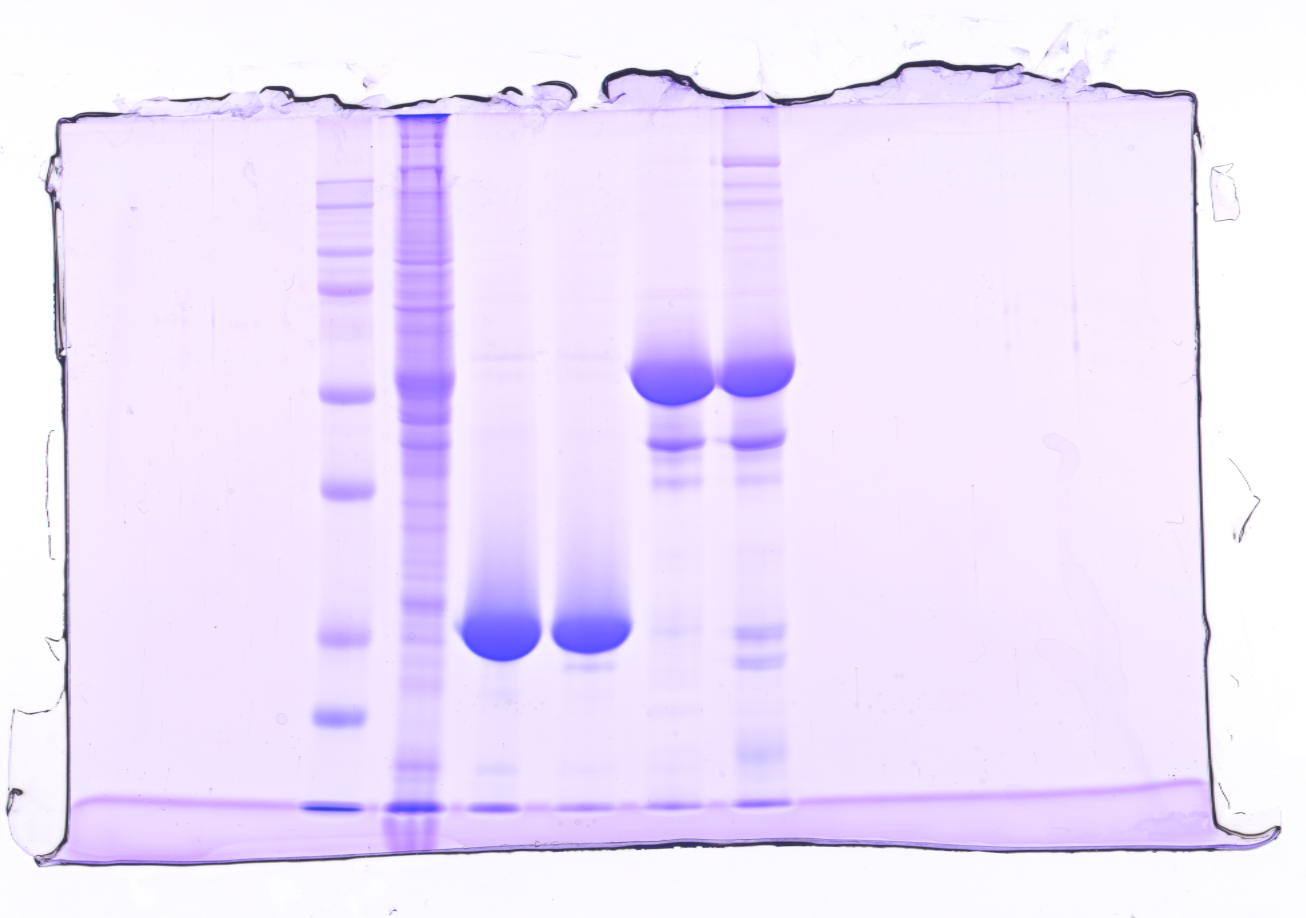

Supplement: Figure 6—figure supplement 1—source data 1. [file elife-83276-fig6-figsupp1-data1.zip › full_unedited_gels_S6A_left.tif]

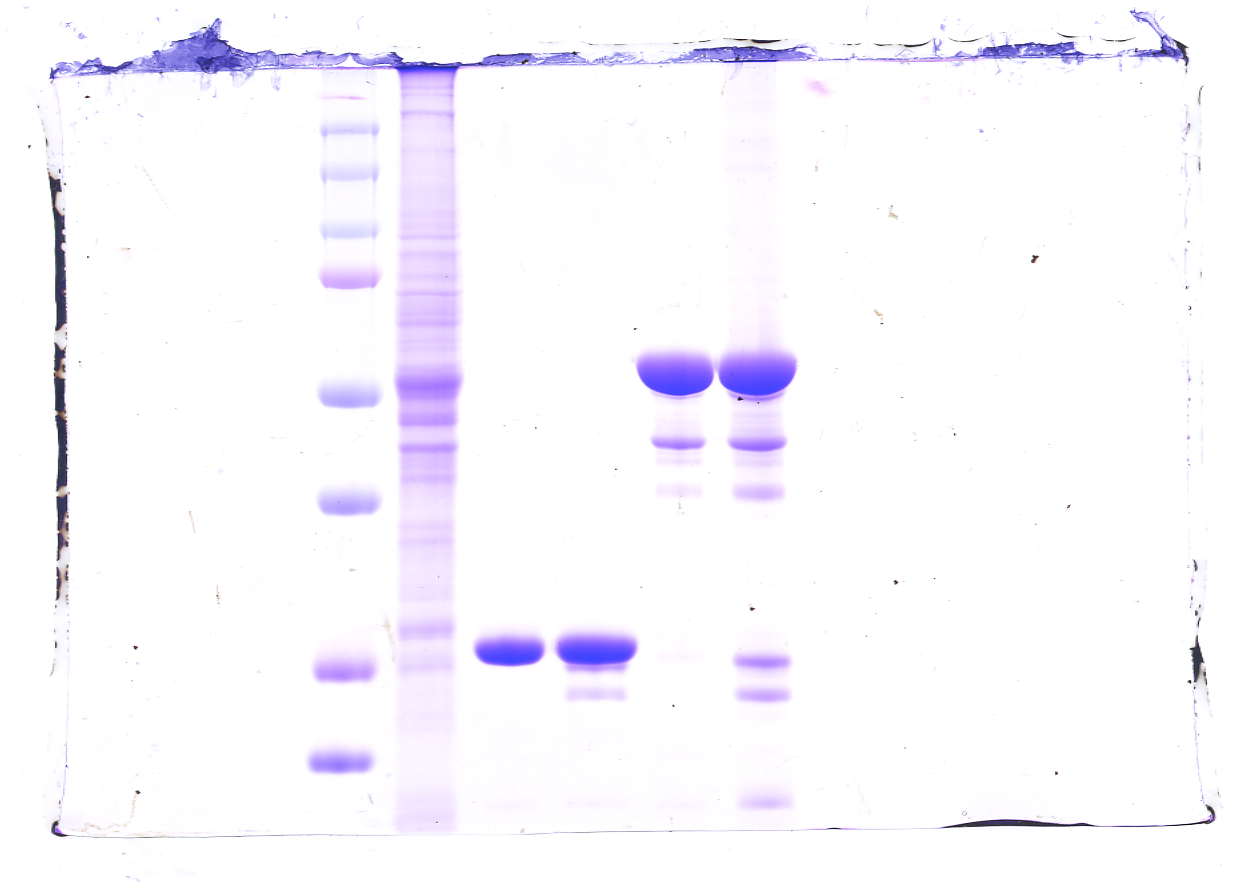

Supplement: Figure 6—figure supplement 1—source data 1. [file elife-83276-fig6-figsupp1-data1.zip › full_unedited_gels_S6A_right.tif]

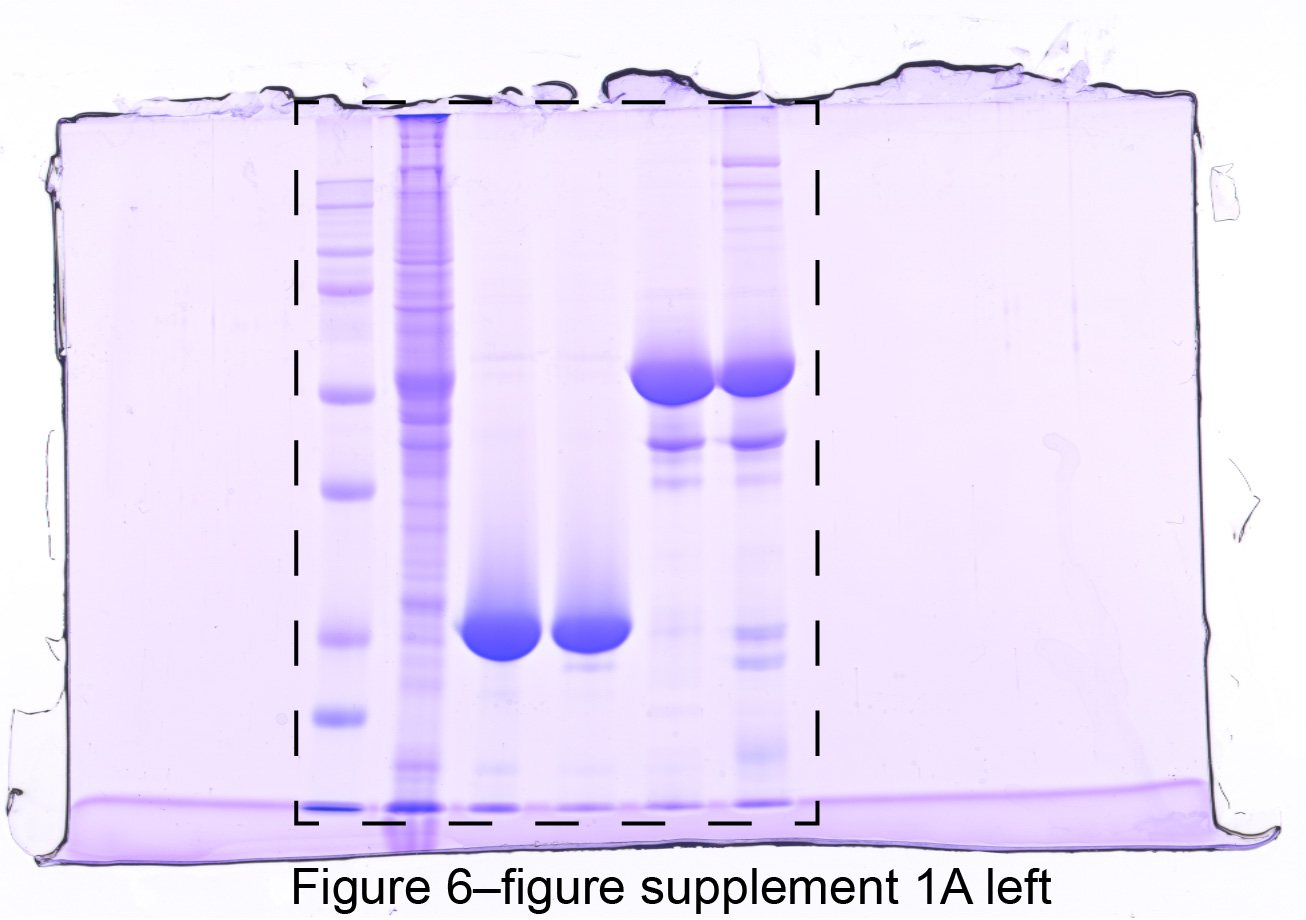

Supplement: Figure 6—figure supplement 1—source data 1. [file elife-83276-fig6-figsupp1-data1.zip › full_unedited_gels_with_label_S6A_left.jpg]

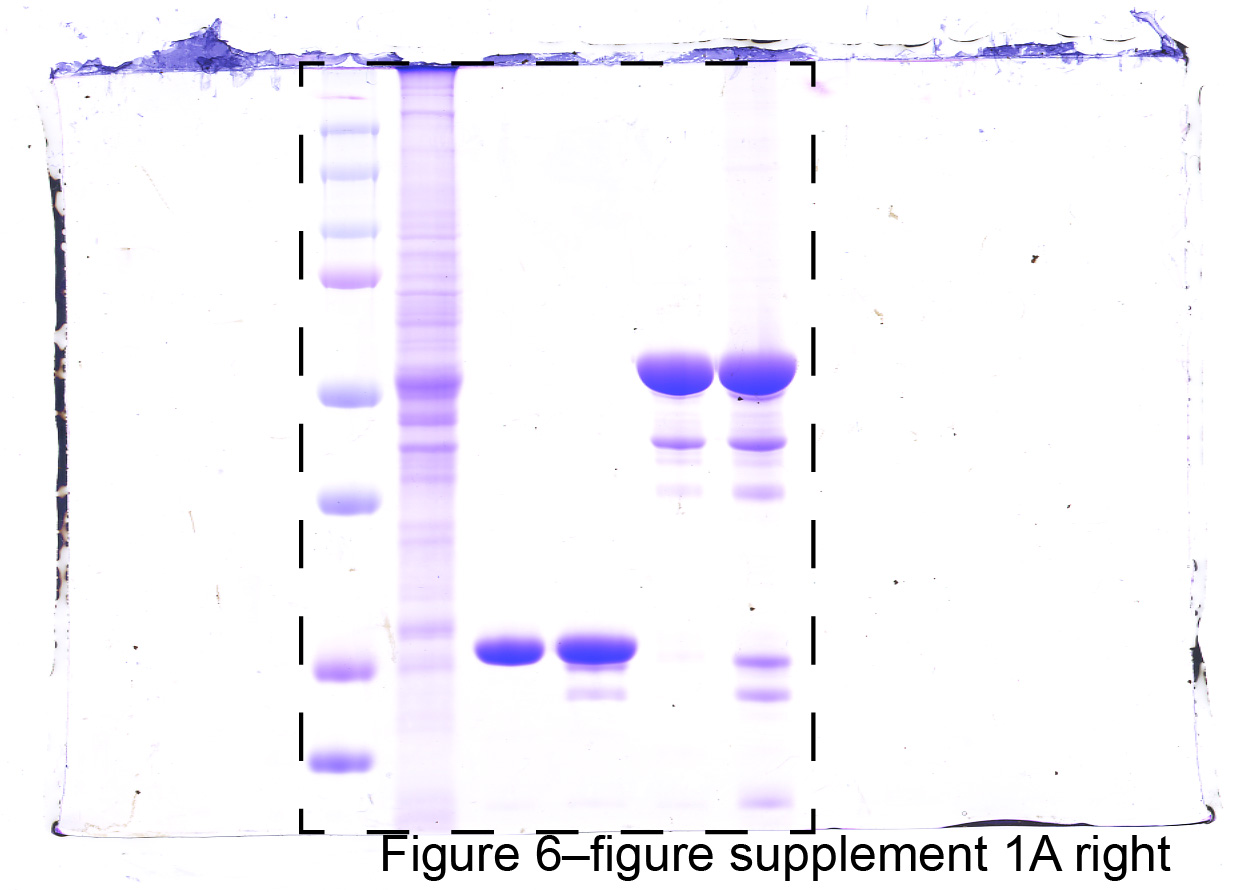

Supplement: Figure 6—figure supplement 1—source data 1. [file elife-83276-fig6-figsupp1-data1.zip › full_unedited_gels_with_label_S6A_right.jpg]

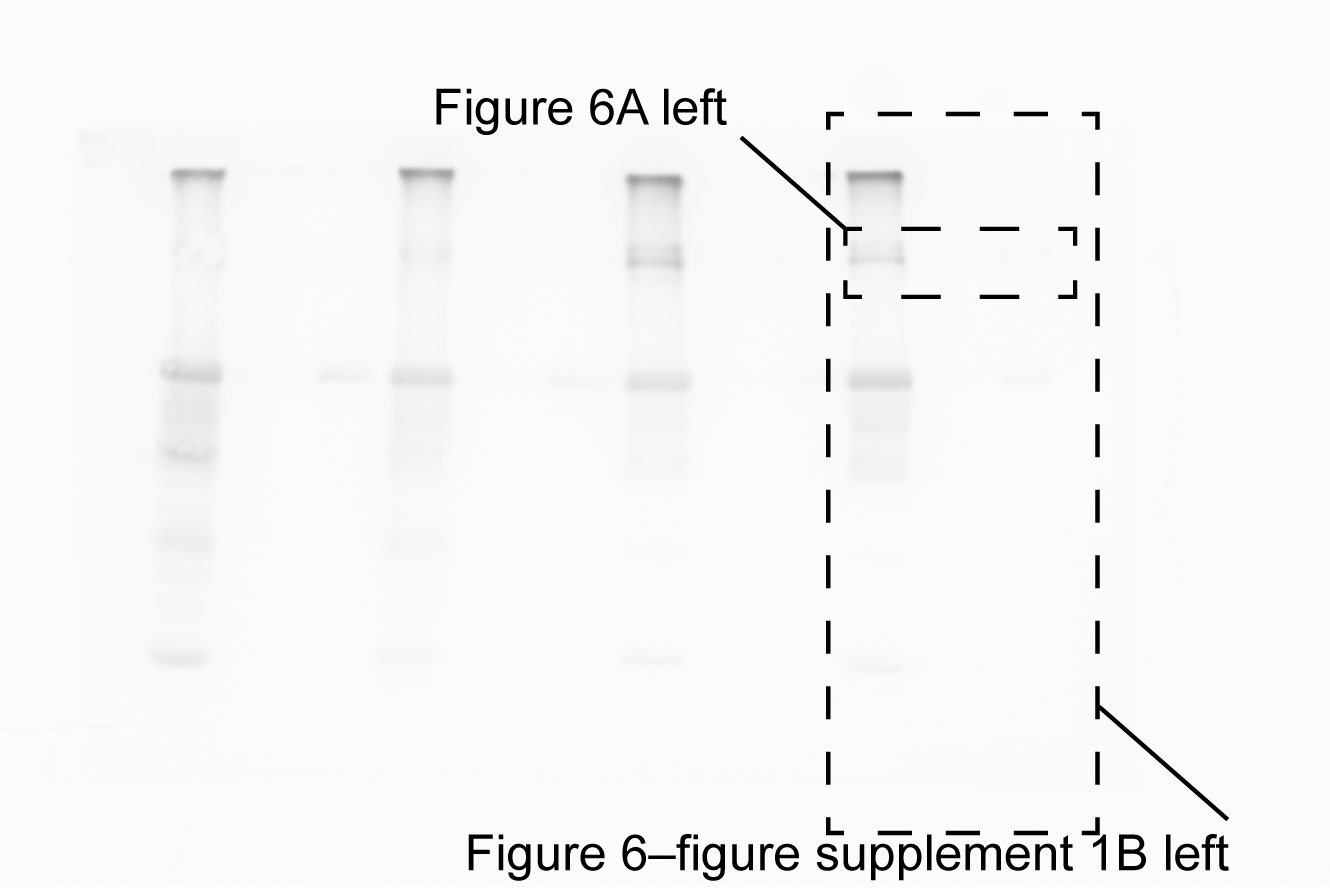

Supplement: Figure 6—figure supplement 1—source data 1. [file elife-83276-fig6-figsupp1-data1.zip › full_unedited_blots_with_labels_S6B_left.tif]
